# Textural, Sensory, and Chemical Characteristic of Threadfin Bream (*Nemipterus* sp.) Surimi Gel Fortified with Bio-Calcium from Bone of Asian Sea Bass (*Lates calcarifer*)

**DOI:** 10.3390/foods10050976

**Published:** 2021-04-29

**Authors:** Ima Wijayanti, Avtar Singh, Soottawat Benjakul, Pornsatit Sookchoo

**Affiliations:** 1 International Center of Excellence in Seafood Science and Innovation, Faculty of Agro-Industry, Prince of Songkla University, Hat Yai 90110, Thailand; imasetianto@gmail.com (I.W.); avtar.s@psu.ac.th (A.S.); 2 Department of Fish Product Technology, Faculty of Fisheries and Marine Science, Diponegoro University, Semarang 50275, Indonesia; 3 Center of Excellence in Bio-Based Materials and Packaging Innovation, Faculty of Agro-Industry, Prince of Songkla University, Hat Yai 90110, Thailand; pornsatit.s@psu.ac.th

**Keywords:** fish bone bio-calcium, threadfin bream, surimi, gel properties, sensory, microstructure

## Abstract

The effects of Asian sea bass (*Lates calcarifer*) bio-calcium (ASBB) at different levels (0, 2, 4, 6, 8, and 10%) (*w*/*w*) on properties of threadfin bream (*Nemipterus* sp.) surimi gel were investigated. ASBB addition increased breaking force and deformation, while reduced expressible moisture content (*p* < 0.05) of surimi gel. L* (lightness), a* (redness), and b* (yellowness) values were increased with augmenting ASBB levels; however, whiteness slightly decreased in surimi gel incorporated with ASBB (*p* < 0.05). Higher likeness scores were noticed in surimi gel containing ASBB, compared to that of the control. However, a slight decrease in the likeness score was noticed in surimi gel with 10% (*w*/*w*) ASBB (*p* < 0.05). Surimi gel added with 8% (*w*/*w*) ASBB possessed the increase in breaking force by 80% from the control and had the highest likeness score. Texture profile analysis of surimi gel added with ASBB showed the improved texture characteristics with coincidentally higher storage modulus of surimi paste. Surimi gel with 8% (*w*/*w*) ASBB had a denser and finer microstructure with higher ash, calcium, and phosphorous contents, compared to the control. Thus, incorporation of bio-calcium up to 8% (*w*/*w*) not only increased mineral content, but also improved textural, sensory, and microstructural properties of surimi gel.

## 1. Introduction

Surimi is a concentrated fish myofibrillar proteins, in which sarcoplasmic protein, lipids, bloods, odorous compounds, and pigment are removed via thorough washing. Surimi generally has a better gel-forming capability than its corresponding mince [1]. Myofibrillar proteins play a major role in the formation of preferable texture of surimi gel. By means of a gelling process, a three-dimensional gel network is developed as a result of the exposure of functional groups of numerous protein molecules, with subsequent inter- or intra-molecular interactions. Several additives were previously employed to enhance gel characteristics of surimi such as egg white [2,3], whey protein concentrate [4,5,6], oxidized phenolic compounds [7,8], coconut husk extract [9], some hydrocolloids [10,11,12], calcium chloride [13], calcium carbonate [14], etc.

The washing and dewatering process in surimi also remove minerals including calcium present in the fish meat. The addition of calcium compounds such as calcium chloride and calcium carbonate could improve the gelling ability of surimi. Calcium chloride together with whey protein concentrate increased gel properties of goatfish surimi [4]. Generally, during setting, calcium ion liberated from the calcium salts activates endogenous transglutaminase (TGase), thereby catalyzing the formation of *ε*-(*γ*-glutamyl) lysine linkages between myofibrillar proteins. Additionally, the formation of calcium-bridges between unfolded negatively charged myofibrillar proteins may assist to strengthen gel texture [15].

Fortification of functional ingredients in the form of minerals and vitamins in foods is commonly accepted for daily intake. The addition of calcium ion in surimi is not only for gel enhancement, but also for fortification of calcium due to the negligible calcium content in surimi [16]. Calcium-fortified products would be beneficial in the improvement of the amounts of calcium intake, especially for milk and dairy products among intolerance population groups [17]. Fish bio-calcium can be fortified into many foods to increase calcium and other minerals such as phosphorus, iron, magnesium, etc. In general, textural characteristics of surimi gel were improved with addition of calcium, with coincidentally increased sensory characteristic [4,14,18,19]. Nevertheless, fish bone bio-calcium might contribute to the grittiness or sandy mouth-feel of surimi gel due to the compact and rigid bio-calcium particles, as long as bio-calcium is still large in size. To mitigate such a drawback, the bio-calcium was successfully developed with the negligible grittiness with fine particles using the simple and cheap method [20]. Surimi incorporated with micro- and nano-scale fish bone had the improved gel property. Calcium ion could be liberated, thus increasing the setting phenomenon and producing a stronger gel network [15,21,22]. Micro- and nano-scale fish bone powders were incorporated into surimi gel in the range of 1–2% (*w*/*w*) and subsequently yielded the surimi gel rich in calcium. To our knowledge, no reports are available for the use of bio-calcium from Asian sea bass as the natural calcium source for improvement of surimi gel and as a calcium supplement. Therefore, the aim of the current study was to elucidate the effect of finely ground defatted and bleached bio-calcium from Asian sea bass (*Lates calcarifer*) bone at different levels on the texture, sensory property, microstructure, and chemical composition of threadfin bream (*Nemipterus* sp.) surimi gel.

## 2. Materials and Methods

### 2.1. Materials

Bio-calcium was produced from the Asian sea bass frame attained from Kingfisher Holdings Co., Ltd., Songkhla, Thailand. Frozen frames were kept in a polystyrene box and transported by a refrigerated truck to International Center of Excellence in Seafood Science and Innovation, Prince of Songkhla University, Hat Yai, Thailand within 1 h. Frames were cut longitudinally and washed using the running water until blood and bone marrow were totally removed. The clean bones were packed in the polyethylene bag and kept at −20 °C before use. Grade B frozen threadfin bream surimi was bought from Chaichareon Marine Co., Ltd. (Pattani, Thailand) and kept at −20 °C for less than 1 month. Moisture, protein contents, and pH of surimi were 77.99% (*w*/*w*), 13.03% (*w*/*w*), and 7.41, respectively. Surimi contained polyphosphate, sorbitol, and sucrose of 0.50, 3, and 3% (*w*/*w*), respectively.

### 2.2. Chemicals

Chemicals with analytical grades used for SDS-PAGE (sodium dodecyl sulfate-polyacrylamide gel electrophoresis) were obtained from Bio-Rad (Hercules, CA, USA). The rest of the chemicals were bought from Sigma (St. Louis, MO, USA).

### 2.3. Preparation of Bio-Calcium

Asian sea bass bio-calcium (ASBB) was produced, as detailed by Wijayanti et al. [20]. Asian sea bass backbones were boiled at 100 °C for 30 min. Boiled bones were further washed using tap water to remove adherent meat. Cleaned bones were added with distilled water using a ratio of 1:4 (*w*/*v*) and autoclaved (high-pressure heated) at 121 °C for 90 min. The autoclaved bones were dried for 24 h at 50 °C using an oven (Memmert UF30, GmbH + Co.K, Schwabach, Germany) and were subjected to a multifunctional grinder (Model1000A, Qingdao, China) to gain the coarse powder. The obtained powder was soaked in hexane with a ratio of 1:10 (*w*/*v*) and mixed using a stirrer (Model RW20.n, IKA-Werke Gmb H&CO. KG, Staufen, Germany) at 150 rpm for 60 min at room temperature (RT, 28−30 °C). Hexane was drained, and samples were left at RT. Afterward, the de-fatted powder was bleached using 2.50% (*v*/*v*) hydrogen peroxide (H_2_O_2_) at a sample/solvent ratio of 1:10 (*w*/*v*), followed by stirring at RT for 60 min. The bleached powder was rinsed for 15 min using tap water and subsequently dried at 50 °C in an oven for 24 h. The dried powder (200 g) was further milled using a ball mill machine (PM 100, Retsch GmbH, Haan, Germany) using 25 grinding balls (20 mm diameter) for 2.50 h at a speed of 200 rpm. The resulting powder was sieved by a sieving machine (EVS1, Endecotts Ltd., London, England) using a 75 µm sieve to obtain a fine powder (ASBB). Particle size of ASBB was 19.72 ± 4.25 µm as determined by a Laser Particle Size Analyzer (LPSA, LS 230, Coulter, CA, USA).

### 2.4. Preparation of Surimi Gel

Surimi gel preparation was performed as per the procedure of Buamard and Benjakul [23]. Frozen surimi was kept at 4 °C for 60 min and further cut into cubes (3 × 3 × 3 cm). Surimi cubes were chopped and added with sodium chloride (NaCl) (2%; *w*/*w*) in a food processor (National Model MK-5080M, Selangor, Malaysia) for 1 min. ASBB powder at 0, 2, 4, 6, 8, and 10% (*w*/*w*) was added, and the final moisture content was adjusted to 80% (*w*/*w*). The mixture was chopped for 30 s with a 5 s rest interval for a total of 4 min. During the mixing process, surimi paste temperature was held under 10 °C. The surimi paste was loaded into a polyvinyl ethylene chloride casing with a diameter of 2.5 cm and a length of 25 cm and sealed. The gel setting was performed in a water bath at 40 °C for 30 min to activate endogenous transglutaminase (TGase). Thereafter, the samples were heated for 20 min at 90 °C to inactivate endogenous TGase and proteases. The samples were further cooled using the iced water for 60 min and kept in a refrigerator (4 °C) overnight. Gel without ASBB was named as “CON”, while those added with 0, 2, 4, 6, 8, and 10% (*w*/*w*) were referred to as “SBC-2”, “SBC-4”, “SBC-6”, “SBC-8”, and “SBC-10”, respectively. Breaking force (B-f), deformation (D-f), expressible moisture content (EMC), color, SDS-PAGE protein pattern, and sensory characteristics of surimi gel were evaluated.

### 2.5. Analyses

#### 2.5.1. Breaking Force and Deformation

Breaking force (B-f) and deformation (D-f) tests were established, as tailored by Sae-Leaw et al. [24], using a texture analyzer (Model TA-XT2i, Stable Micro Systems, Surrey, UK). The spherical plunger (diameter: 5 mm) was used at a compression velocity of 0.10 cm/s. Before analysis, surimi gel samples having a diameter of 2.50 cm and a height of 2.50 cm were prepared and allowed to stand at RT for 1 h.

#### 2.5.2. Expressible Moisture Content (EMC)

EMC was measured as per the method of Balange and Benjakul [7] with some modifications. Surimi gels were cut to get a 5 mm thickness and weighed (*A*). Samples were then put between Whatman paper No. 4 (2 pieces) on the top and 3 pieces at the bottom of the samples. *A* standard weight (5 kg) was located on the top and left for 2 min. The pressed gel samples were weighed (*B*) after being taken off from the papers. EMC was measured by gravimetry using this following calculation,

(1) $$\mathrm{EMC}\;(\%)=\frac{A-B}{A}\;\times \;100$$

#### 2.5.3. Color

The color of surimi gel was analyzed using Hunterlab (ColorFlex, Hunter Associates Laboratory, Reston, VA, USA). Lightness (L*), redness/greenness (a*), and yellowness/blueness (b*) were measured by the reflection method. Whiteness index was computed as guided by NFI [25] using the subsequent equation,

Whiteness Index = 100 − [(100 − L*)^2^ + (a*)^2^ + (b*)^2^]^(1/2)^ (2)

#### 2.5.4. Sodium Dodecyl Sulfate-Polyacrylamide Gel Electrophoresis (SDS-PAGE)

Protein patterns of surimi gels and pastes were analyzed by SDS-PAGE following the method of Singh and Benjakul [26]. Samples (2 g) were firstly mixed with 5% (*w*/*v*) SDS solution (85 °C) and homogenized using a homogenizer (IKA Labortechnik, Selangor, Malaysia) at 10,000 rpm for 1.5 min. The mixture sample was heated for 1 h at 85 °C. The samples were centrifuged at 7000× *g* for 15 min and afterward, the protein content of each sample was analyzed by the Biuret method using bovine serum albumin as the standard [27]. The protein content of each sample was then adjusted to 6 mg/mL. Subsequently, samples were mixed with sample buffer containing 10% (*w*/*v*) β-mercaptoethanol, 4% (*w*/*v*) SDS, 20% (*w*/*v*) glycerol, and 0.001% (*w*/*v*) bromophenol blue at a ratio of 1:1 (*v*/*v*) and boiled at 85 °C for 30 min before being loaded. The polyacrylamide gels were prepared using 7.50 or 10% (*w*/*v*) running gels and 4% (*w*/*v*) stacking gel. Samples (15 µg protein) were loaded and subjected to electrophoresis at a 15 mA current per gel, using a Mini Protein II unit (Bio-Rad Laboratories, Inc., Richmond, CA, USA). Standard markers were also used. After separation, the proteins were stained for 24 h using 0.05% (*w*/*v*) Coomassie Blue R-250 in 50% (*v*/*v*) methanol and 7.5% (*v*/*v*) acetic acid, followed by de-staining using the mixture of 30% (*v*/*v*) methanol and 10% (*v*/*v*) acetic acid for 6 h. Band intensity of myosin heavy chain (MHC) with a molecular weight around 200 kDa of surimi paste and gel appearing in 7.50 or 10% (*w*/*v*) running gel was determined by an image analysis using a Model GS-700 Imaging Densitometer (Bio-Rad Laboratories, Hercules, CA, USA) with the aid of molecular Analyst Software version 1.4.

#### 2.5.5. Acceptability

The sensory analysis was performed following the NIH guidelines [28]. Surimi gel samples were cut into a bite size having 3 cm diameter and 1 cm thickness and conditioned for 30 min at RT. One hundred un-trained panelists (61 female and 39 males with the age of 18–55 years) were the students and staffs at the Faculty of Agro-Industry, who had no seafood allergy and often consumed surimi products. The panelists were asked to test for texture, color, odor, flavor, taste, sandy mouth-feel, and overall liking of gel samples using a 9-point hedonic score (from 1, extremely dislike to 9, extremely like) [29]. Distilled water was served for rinsing the mouth between the samples. Panelist were also provided with plain crackers to neutralize the taste, odor, and flavor of surimi samples.

### 2.6. Characterization of Selected Surimi Gel in Comparison to Control Gel

ASBB at the selected level was added in surimi, and the gel was prepared as mentioned above (Section 2.4). The control gel (surimi without bio-calcium addition) was also prepared. Furthermore, texture profile, Scanning Electron Microscope-Energy Dispersive X-ray (SEM-EDX), rheological properties, proximate compositions, calcium, and phosphorus contents were analyzed.

#### 2.6.1. Texture Profile Analysis (TPA)

TPA of selected surimi gels were examined following the condition described by Gani et al. [30]. Surimi gels with a 2.50 cm diameter were taken for the TPA. Surimi gel samples were subjected to two-cycle pressure at 50% strain using a texture analyzer equipped with a P/50 TPA cylinder probe. Operation was done at a test speed of 5 mm/s. Hardness, cohesiveness, springiness, chewiness, gumminess, adhesiveness, and resilience were determined.

#### 2.6.2. Scanning Electron Microscope-Energy Dispersive X-ray (SEM-EDX)

The microstructure and elemental distribution of surimi gels without and with ASBB were analyzed using a Field Emission Scanning Electron Microscope (Apreo, FEI, Eindhoven, The Netherlands). The visualization was done at 20 kV with a magnification of 10,000× as per the condition described by Gani et al. [30].

#### 2.6.3. Proximate Compositions

The determination of proximate compositions including protein, fat, and ash using analytical Nos. 950.46, 920.153, and 960.39, respectively, was done as described by AOAC [31]. Carbohydrate content was determined by considering the rest of constituents. The content was reported as dry weight basis.

#### 2.6.4. Calcium and Phosphorus Contents

Calcium and phosphorus contents were quantified using an inductively coupled plasma optical emission spectrometer (ICP-OES) (Avio500, Perkin Elmer Instruments, Waltham, MA, USA). The sample (0.1 g) was mixed with 8 mL of concentrated nitric acid and 4 mL of hydrofluoric acid. The mixed sample was digested by a microwave digester (1650 W) using three-step heating. The first step of heating was done for 20 min at 150 °C, followed by 25 min at 175 °C, and lastly 35 min at 200 °C. The digested sample was diluted 100 times before analysis. ICP-OES was operated at wavelengths of 214.914 nm, power of 1400 KW, auxiliary gas flow rate of 0.5 L/min, atomizer flow rate of 0.5 L/min, plasma flow rate of 18 L/min, and observation distance of 15 mm. Argon was used. Concentrations of the standard used were 0.2, 0.5, 1, 1.5, 2, and 3 mg/L.

#### 2.6.5. Rheological Properties

An oscillatory temperature sweep was used to analyze the rheological behavior of the surimi paste. Rheological determination was carried out with a Rheometer (HAAKE RheoStress1, Themo Fisher Scientific, Karlsruhe, Germany) with a cone and plate geometry. Samples were swept with a 1 Hz constant strain amplitude at temperatures from 20 to 90 °C using a heating rate of 1 °C/min [32].

### 2.7. Statistical Analysis

A completely randomized design (CRD) was used for the entire study. Analysis of variance was done for all the data. The Duncan’s multiple range test was performed for means comparison. The selected sample and control were statistically compared using a T-test. Statistical Package for Social Science (SPSS) (SPSS 16.0 for windows, SPSS Inc., Chicago, IL, USA) was used for statistical analysis with significance level of 0.05. All experiments were done in triplicate.

## 3. Results and Discussion

### 3.1. Characteristics of Surimi Gel as Affected by ASBB at Different Concentrations

#### 3.1.1. Breaking Force and Deformation

**Figure 1:**
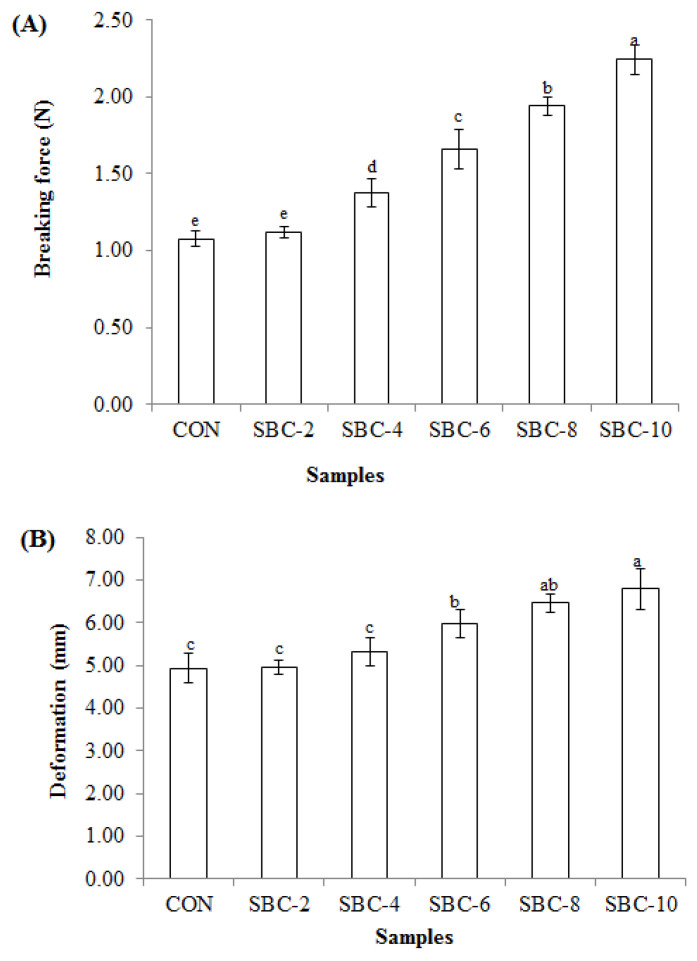
Breaking force (**A**) and deformation (**B**) of threadfin bream surimi gel added with different levels of ASBB. Different lowercase letters denote significant differences (*p* < 0.05). Values represent mean ± SD (n = 3). CON: Control (without addition of ASBB). SBC-2, SBC-4, SBC-6, SBC-8, and SBC-10: surimi gel added with ASBB (SBC) at 2, 4, 6, 8, and 10% (*w*/*w*), respectively.

Breaking force (B-f) and deformation (D-f) of threadfin bream surimi gel at different concentrations of Asian sea bass bio-calcium (ASBB) are presented in Figure 1A,B, respectively. ASBB significantly affected the B-f and D-f of surimi gel (*p* < 0.05). The lowest B-f was obtained in the CON, and the highest value was obtained for SBC-10 (*p* < 0.05). Both B-f and D-f increased for all surimi gels with ASBB addition. The B-f of SBC-2, SBC-4, SBC-6, SBC-8, and SBC-10 samples were augmented by 5, 28, 51, 80, and 108%, respectively, compared to that of the CON. However, ASBB addition of more than 10% (*w*/*w*) drastically reduced B-f and D-f (see Supplementary Materials Figure S1).

In general, the increases in B-f were more likely due to endogenous TGase, a crosslinker of MHC via the formation of *ε*-(*γ*-glutamyl)-lysine linkage during setting at 40 °C. Chanarat, Benjakul and H-Kittikun [33] reported that the endogenous TGase plays an important role in myofibrillar crosslinking. The lower B-f was noticed in surimi gel prepared from different fish species in the presence of EDTA, a metal chelator, which acts as TGase inhibitor. In the current study, calcium ion (Ca^2+^) released from ASBB during setting at 40 °C was able to increase the TGase activity, thus yielding a gel with higher B-f. According to Yongsawatdigul et al. [34], during grinding the surimi in the presence of NaCl, myofibrillar proteins were solubilized, resulting in partial unfolding of actomyosin, in which reactive groups could be exposed for crosslinking mediated by TGase. The Ca^2+^ from ASBB enhanced or activated the activity of endogenous TGase, a crosslinker of MHC. Moreover, calcium ion from ASBB during setting might lead to the unfolding of MHC, which favored a TGase-mediated reaction and enhanced the level of hydrophobic-hydrophobic interaction [15]. Yongsawatdigul et al. [34] reported that endogenous TGase of threadfin bream surimi was activated by Ca^2+^ ion from calcium chloride (CaCl_2_) during setting at 40 °C, resulting in higher B-f than that of the control. CaCl_2_ and calcium carbonate (CaCO_3_) as the source of Ca^2+^ ion in surimi from bigeye snapper [19], goatfish [4], carps [35], etc. Ca^2+^ ion from micro- and nano-scale fish bone was reported to increase endogenous TGase activity of silver carp [15] and Alaska pollock [22] surimi. TGase activity of surimi was generally varied, depending on the amount of Ca^2+^ ion and fish species [18]. After setting, oxidations of sulfhydryl groups with the accompanied disulfide bond formation occurred as induced by rising temperatures during heating [8,35]. Instability of hydrogen bonds during heating unfolds some proteins. Hence, hydrophobic amino acids were exposed, causing a higher hydrophobic interaction [19]. During cooling, a hydrogen bond could be formed between proteins, which stabilize the β-structure and the α-helix of partially denatured and native proteins and yield a stronger gel network [36].

For D-f, values of all gels were in the range of 4.94−6.79 mm, regardless of ASBB addition. The highest D-f was obtained in the SBC-10 sample; however, no difference was noticed between the SBC-8 and SBC-10 samples (*p* > 0.05). This non-significant difference might be due to protein dilution with the ASBB addition. Dilution of myofibrillar proteins by ASBB might help lower the excessive interaction between protein chains, leading to lower elasticity or extensibility. Moreover, the higher amount of ASBB added could lower the formation of a strong bond such as a disulfide bond. In the present study, the similar formation of the elastic gel was attained between the SBC-8 and SBC-10 samples (*p* > 0.05). On the other hand, B-f was increased with the addition of ASBB. Despite activation of TGase, ASBB might act as a gel filler, which could increase the B-f. Moreover, Ca^2+^ ion was also previously found to destroy the α-helical structure of myosin, leading to enhancement of hydrophobic interactions and improvement of textural properties [22]. Yin et al. [15] reported that Ca^2+^ ion from fish bone powder tended to form a salt-bridge among myofibrillar proteins with a negative charge, which may assist to improve gel characteristics. Although the surimi protein was lessened, the ASBB could play a major role in strengthening the gel network as indicated by the increased B-f, mainly by a filling effect.

An ASBB higher than 10% resulted in a drastic reduction in B-f and D-f. This was more likely associated with the reduction in myofibrillar proteins (dilution effect). Myofibrillar proteins, particularly MHC, play a major role in gelation of surimi. Similarly, Yin and Park [22] noticed the formation of gel with lower textural properties when the concentrations of nano-scaled fish bone were increased.

#### 3.1.2. Expressible Moisture Content (EMC)

In general, EMC represents the water holding capacity (WHC) of surimi gel. A lower EMC indicates more water retained or bound with the gel matrix [13]. The EMC of surimi gels containing ASBB at different concentrations is displayed in Table 1. All surimi gels incorporated with ASBB exhibited lower EMC than the CON (*p* < 0.05). Nevertheless, SBC-2, SBC-4, and SBC-6 samples had a similar EMC (*p* > 0.05). The lowest EMC was attained for SBC-10 (*p* < 0.05). Nevertheless, similar EMC was noted for SBC-6 and SBC-8 (*p* > 0.05). The EMC of surimi gel was decreased by 16.99−28.29% with an addition of 2−10% (*w*/*w*) ASBB. Ca^2+^ ion in ASBB tended to increase the WHC of surimi gel as ascertained by the lowered EMC than the CON. Although protein content was slightly reduced, more water entrapped in the gel network in the presence of ASBB was obtained. Regardless of initial moisture content (80%; *w*/*w*), the released or bound water varied depending on the ability of the network to hold water. In addition, ASBB distributed inside the gel might help trap the water within the gel as indicated by the lowered EMC. During setting, Ca^2+^ ion more likely increased the gel-forming capability via formation of non-disulfide covalent bonds. Therefore, more water could be imbibed or entrapped in the gel matrix [4]. This coincided with higher B-f and D-f of surimi gels incorporated with ASBB (Figure 1). Benjakul et al. [4] noticed that the EMC of goat fish kamaboko gel with CaCl_2_ was lower than the CON, indicating higher WHC of the resulting gel. In addition, WHC of silver carp surimi gel with setting increased when micron fish bone was added [15].

#### 3.1.3. Color

Color parameters of surimi gel with ASBB added at varying concentrations are shown in Table 1. ASBB at various concentrations had varying impacts on L*, a*, b*, and whiteness index of the resulting gel. The highest L*, a*, and b* values were found in SBC-10 sample (*p* < 0.05). However, no different lightness (L*) was observed between SBC-8 and SBC-10 (*p* > 0.05). The whiteness index was slightly decreased when ASBB more than 4% (*w*/*w*) was incorporated (*p* < 0.05). The lowest whiteness was noticed in the SBC-10 sample (*p* < 0.05).

An increase in lightness (L*) of surimi gel might be due to light scattering by ASBB particles. This led to the increased lightness of gel, particularly at higher levels used. The increases in a* and b* values with augmenting ASBB concentrations were more likely caused by color characteristics of the ASBB powder. Wijayanti et al. [20] reported that lightness (L*), redness/greenness (a*), yellowness/blueness (b*) and whiteness of Asian sea bass backbone bio-calcium were 94.50, 0.14, 5.63, and 92.27 respectively. The whiteness of surimi gel slightly decreased with an increase in ASBB concentration. This might be caused by the milky white color of fishbone bio-calcium. During bleaching of fish bone with the help of strong oxidizing agents, lipid and protein retained in bone underwent oxidation, and the carbonyl was generated. Subsequently, carbonyls reacted with the free amino group, leading to the Maillard reaction [20]. The result was supported by the increasing lipid oxidation of fish bio-calcium during preheat-treatment and bleaching as evidenced by the increased TBARS value [20]. Whiteness slightly decreased in silver carp [15] and Alaska pollock [22] surimi gel with the fishbone addition. Khoder et al. [37] found that whiteness of tofu was slightly reduced after being incorporated with nano-fishbone. Higher whiteness (79.18–80.80) was observed in this study, compared to surimi gel added with micron fishbone powder from silver carp (47.60−56.50) [15] and Alaska pollock (59.93−73.72) [22]. This might be governed by the different species of surimi and fishbone bio-calcium used. Therefore, the color of surimi gel was slightly affected by ASBB and levels added.

#### 3.1.4. Protein Pattern

**Figure 2:**
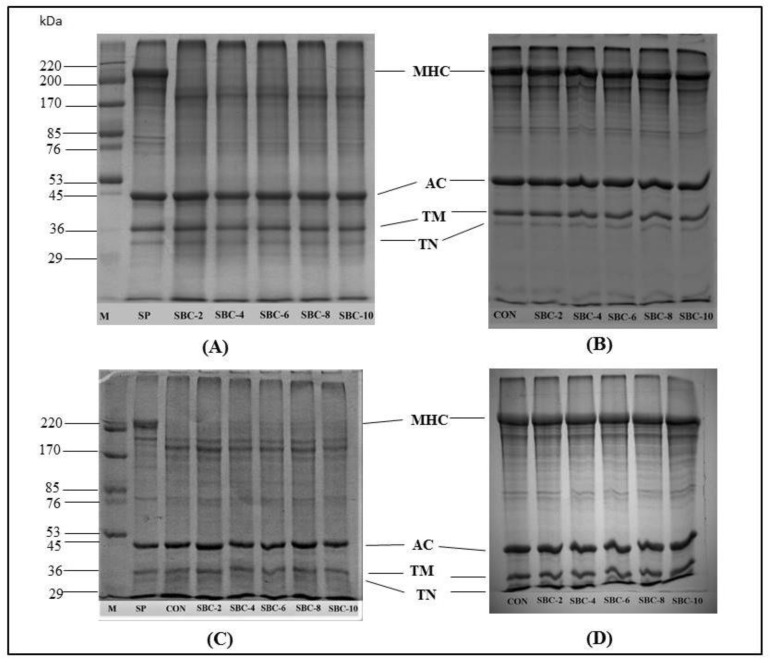
Protein patterns of threadfin bream surimi gel and paste added with different levels of Asian sea bass bio-calcium. Caption: See Figure 1. Surimi gel (**A**) and paste (**B**) separated using 10% running gel. Surimi gel (**C**) and paste (**D**) separated using 7.5% running gel. Protein concentration of each well: 15 µg; M: marker with molecular weights of 220, 170, 85, 76, 53, 45, 36, and 29 kDa; SP: Surimi paste; MHC: myosin heavy chain; AC: actin; TM: tropomyosin; TN: troponin.

Protein patterns of surimi paste, and gel added with ASBB at varying levels, are displayed in Figure 2. The MHC and actin were dominant proteins in all the samples. MHC, actin, tropomyosin, and troponin bands of threadfin bream surimi paste, and gel were detected at the molecular weight of 200.10, 46.70, 35.60, and 30.60 kDa, respectively (Figure 2). Supreetha et al. [38] reported that the MHC band of threadfin bream fish mince was observed with a molecular weight of 205.00 kDa. The actin band of several surimi gels were noticed in the molecular weight range of 45−55 kDa [34,39,40]. Suarezet et al. [41] reported that tropomyosin and troponin bands of surimi from weakfish were observed at the molecular weight of 36 and 31 kDa, respectively.

The MHC band of the CON gel and gels added with different levels of ASBB were totally disappeared, while a large MHC band was observed in the paste (Figure 2A,C). The MHC was still crosslinked together by TGase in the presence of ASBB, indicating that ASBB had no negative impact on protein polymerization during heat-induced gelation. The band intensity of proteins with molecular weights of 174 kDa (lower than MHC bands) including CON, SBC-2, SBC-4, SBC-6, SBC-8, and SBC-10 were 18.27, 17.53, 17.11, 16.57, 15.75, and 14.77%, respectively. Those proteins might be the degradation products of myofibrillar proteins induced by indigenous proteases during setting and heating. Yongsawatdigul et al. [34] also observed the disappearance of the MHC of threadfin bream surimi gel caused by proteolysis during setting at 40 °C. During setting at 40 °C, MHC polymerization took place, as ascertained by the appearance of polymerized protein with a high molecular weight band on the top of the gel (at the base of wells) [42]. A greater decrease in the MHC band intensity was observed after two-step heating [19,39]. Decreases in MHC intensity could be associated with the *ε*-(*γ*-glutamyl) lysine crosslink formation during setting mediated by endogenous TGase, which could be activated by Ca^2+^ ion liberated from bio-calcium [15]. Furthermore, Yin and Park [22] demonstrated that Ca^2+^ ion from micron fishbone in Alaska pollock surimi could increase TGase activity and cause a conformational alteration. TGase induced the formation of isopeptide bonds between glutamine and lysine residues in proteins, thus promoting both intra- and intermolecular covalent crosslinks [43]. The amount of isopeptide bond during setting correlates with the increase in gel strength. The content of the isopeptide bond, crosslinking of MHC, and gel strength increased with augmenting setting time [44]. This favored the crosslinking of MHC induced by TGase as well as the hydrophobic-hydrophobic interaction. No differences in the band intensity of actin, tropomyosin, and troponin among all samples were observed between all the gel samples. In general, actin and tropomyosin were not the preferred substrate for TGase and were resistant to proteolysis induced by heat stable proteases [13].

When the surimi gel samples were separated using 7.5% (*w*/*v*) running gel (Figure 2A,B), different relative distances (Rf) were observed in comparison to 10% (*w*/*v*) running gel (Figure 2C,D), mainly due to different pore sizes. A pore size of 7.50 % (*w*/*v*) running gel was larger than 10% (*w*/*v*). MHC band in 7.5% (*w*/*v*) running gel of the CON, SBC-2, SBC-4, SBC-6, SBC-8, and SBC-10 totally disappeared, when compared with that of paste. However, the band intensity of the protein bands appearing below MHC bands, which was presumed to be a degradation product during setting caused by indigenous proteases, were 17.55, 17.19, 16.47, 16.35, 16.18, and 15.97%, respectively. Generally, protein patterns of surimi gel with a lower molecular weight were clearly observed using 10−12.5% (*w*/*v*) running gel [39,45,46], whereas a MHC band with a high molecular weight (200kDa) was clearly noticed in 7.5% (*w*/*v*) running gel [47].

For protein patterns of surimi paste (SP) samples added with ASBB at different levels (Figure 2B,D), no differences in protein patterns between surimi pastes without or with ASBB were observed when examined using both 10.00 and 7.50% (*w*/*v*) running gel (Figure 2B,D, respectively). The result was reconfirmed by the similar band intensities of CON, SBC-2, SBC-4, SBC-6, SBC-8, and SBC-10 (14.05, 14.02, 14.03, 14.04, 14.03, and 14.05%, respectively), when studied using 10% running gel. Similarly, for 7.5% running gel, no differences in band intensities for CON, SBC-2, SBC-4, SBC-6, SBC-8, and SBC-10 were noticed (16.08, 16.05, 16.08, 16.06, 16.07, and 16.05%, respectively). The results suggested that proteins, particularly MHC, did not undergo crosslinking in the paste (without setting or heating), regardless of levels of ASBB added.

#### 3.1.5. Acceptability

Acceptability is important for products incorporated with fish bio-calcium, mostly due to sandy mouth-feel, leading to rejection by consumers. ASBB was produced by high pressure heating (autoclaving) to soften the bone and reduce grittiness, in which softer texture could be achieved. The level of bio-calcium should be chosen, not only because of improvement of textural property but also enhancement of acceptability to consumers. The likeness score of the CON and gel added with ASBB at different levels is shown in Table 2. ASBB yielded different effects on texture, odor, sandy mouth-feel, and overall likeness (*p* < 0.05).

The highest texture likeness score was found for SBC-8; however, no differences between SBC-8 and SBC-10 were found (*p* > 0.05). The lowest odor likeness score was found for the CON (*p* < 0.05). For the sandy mouth-feel likeness score, the lower sandy likeness score was found for CON, SBC-2, SBC-4, and SBC-6 by the panelists. Nevertheless, the higher overall likeness score was observed in SBC-8 compared to SBC-10 (*p* < 0.05). No differences among color, flavor, and taste were noticed among the samples, regardless of the levels of ASBB incorporated (*p* > 0.05). The higher texture likeness score in SBC-8 and SBC-10 was in accordance with the higher B-f and D-f (Figure 1). The odor likeness score was increased with increasing ASBB levels. Some fishy odor might be reduced by ASBB, which was more likely due to its ability to entrap volatile compounds of surimi gel such as trimethylamine, 3-methyl-butanal, 2-methyl-butanal, 3-hydroxy-2-butanone, 3-methyl-1-butanol, and 2-methyl-1-butanol. These compounds are commonly found in fish flesh and mainly related to fishy odor [48]. Hence, surimi gel with the ASBB addition was preferred by the panelists. All panelists could not detect a sandy mouth-feel taste of the surimi gel with ASBB addition up to 8% (*w*/*w*). It was noted that the SBC-8 sample showed the highest sandy mouth-feel likeness score, representing the lowest sandiness and grittiness detected. The well-developed gel network for the SBC-8 sample could entrap ASBB inside the network effectively, leading to the masking of the particle as ascertained by the lower sandiness. The lowest likeness score of sandy mouth-feel was attained for the SBC-10 sample (*p* < 0.05), indicating more sandiness or grittiness detected. Slight sandy mouth-feel was detected by some panelists. However, the degree of grittiness was not severe. Wijayanti et al. [20] found that Asian sea bass bone bio-calcium had a particle size of 16.71 µm. Yin and Park [22] documented that no sandy mouth-feel was detected in surimi gel incorporated with fish bone powder that had a particle size smaller than 150 µm. The overall likeness score showed that SBC-8 was the most preferred surimi gel. The addition of 8% (*w*/*w*) ASBB (SBC-8) increased textural and acceptability of surimi gel. Therefore, ASBB at 8% (*w*/*w*) was selected for further characterization, compared to the CON.

### 3.2. Characteristics of the Selected Surimi Gel Added with ASBB Compared to the Control

#### 3.2.1. Texture Profile Analysis

TPA parameters such as hardness, adhesiveness, and cohesiveness have been commonly used for studies of rheological properties and sensory parameters of several foods [49]. TPA parameters of surimi gel without ASBB (CON) and gel added with 8% ASBB (SBC-8) are presented in Table 3. All of the TPA parameters of the CON were different from those of the SBC-8 sample (*p* < 0.05), except springiness, in which no difference was detected (*p* > 0.05).

Hardness was formerly described as “the energy necessary to gain a given deformation” and ordinarily assessed in the first certain bites of oral tests, causing a structural damage of food [50]. The SBC-8 sample had higher hardness than the CON (*p* < 0.05). This higher hardness was in line with the greater B-f (Figure 1). Higher adhesiveness was observed for SBC-8, compared to the CON (*p* < 0.05). Nevertheless, similar springiness was noted between both samples (*p* > 0.05). The SBC-8 sample was generally more elastic than the CON. Adhesiveness and springiness were originally described as elasticity [51]. Nishinari et al. [50] explained cohesiveness as the force of the inside bonds governing the structure of product. The cohesiveness of the SBC-8 sample was higher than the CON, which might be due to the stronger bonds formed between protein chains via non-disulfide covalent bonds as a result of TGase activity induced by Ca^2+^ ion from ASBB. This crosslinking results in the formation of myosin polymers with a coincidental decrease in MHC monomers [52]. Gumminess is the force needed to collapse semi-solid samples to a suitable state of swallowing [53]. The SBC-8 sample possessed higher gumminess than the CON (*p* < 0.05). Similarly, chewiness of SBC-8 was greater than the CON (*p* < 0.05). The chewiness is described as the force to chew the solid sample to a stable state for swallowing (springiness × gumminess) [52]. Higher resilience was observed in the SBC-8 sample, compared to the CON (*p* < 0.05). Resilience used to measure how the sample restores from deformation in terms of both force and speed [54]. Overall, the SBC-8 sample showed the better textural properties than the CON, reconfirming the advantage or benefit of ASBB addition at an appropriate level (8%).

#### 3.2.2. Visco-Elastic Properties

**Figure 3:**
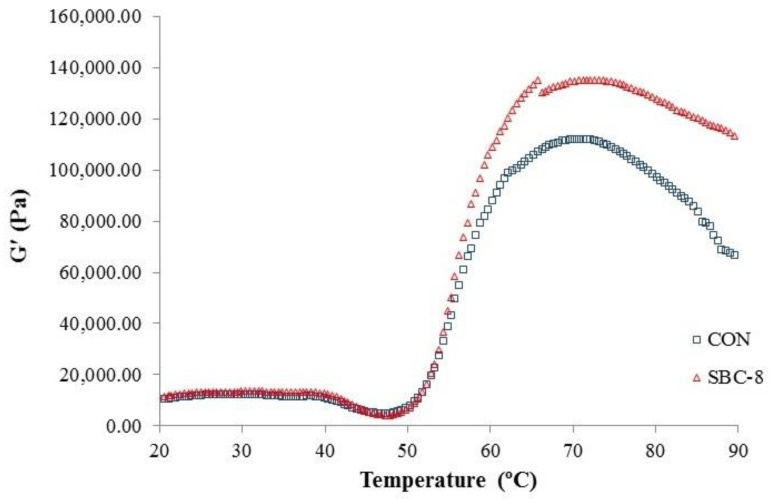
Elastic modulus (G′) of threadfin bream surimi paste without (CON) and with 8% Asian sea bass bio-calcium (SBC-8).

Elastic modulus (G′) of the CON and the SBC-8 sample during the transformation from sol to gel as a function of temperature is illustrated in Figure 3. A similar pattern of Gʹ between both samples was detected; however, a higher Gʹ value was found in the SBC-8 sample. For G′ reflecting an elastic component of the CON and SBC-8 sample, it increased from 10.59 × 10^3^ Pa to 12.18 × 10^3^ Pa and from 11.44 × 10^3^ Pa to 13.46 × 10^3^ Pa, respectively at a temperature of 20–30 °C. At temperatures of 31–47 °C, G′ of the CON and SBC-8 sample decreased and achieved the minimum value of 4.98 × 10^3^ Pa and 4.20 × 10^3^ Pa, respectively. Gel strengthening took place as ascertained by the second increase in Gʹ, starting at 48 °C and reaching the maximum at 72 °C. Maximum Gʹ was 11.21 × 10^4^ Pa and 13.53 × 10^4^ Pa for the CON and SBC-8, respectively. At the final heating stage (90 °C), G′ decreased for the CON and SBC-8 with values of 6.63 × 10^4^ Pa and 10.89 × 10^4^ Pa, respectively.

The slight increase at the first stage (20–30 °C) might be associated with the “salt bridge” formation by Ca^2+^ ion liberated from fishbone bio-calcium. This could favor a reaction with partially unfolded and dissolved protein molecules after chopping [22]. A slight decrease in G′ occurred at 31–47 °C, suggesting the weakening of the protein network in the paste, more likely caused by heat-activated proteases [9]. Those alterations could also be caused by the partial denaturation of muscle proteins [30]. Yin and Park [55] reported that a slight decrease in G′ at a temperature lower than 45 °C on Pacific white surimi paste added with nano fishbone might be due to coil alteration of myosin and possible degradation of the semi gel-like protein network. This resulted in a large increase in fluidity. The sharp increase was denoted at 48–72 °C, that myofibrillar proteins began to form a gel matrix [32]. In this stage, the denatured proteins underwent aggregation and formed the gel matrix with the ability to hold the water [55]. The second drop in Gʹ occurred at the final heating stage (73–90 °C), reflecting that the SBC-8 sample had a higher Gʹ than the CON. Singh et al. [9] found that Gʹ of goatfish surimi paste added with microbial transglutaminase (MTGase) and coconut husk extract decreased at 80 °C. The high temperature more likely destroyed hydrogen bonding and weakened the developed gel. Moreover, the strong gel formed in this stage resulted in the slipperiness of the gel between the plate and cone, leading to the decreased G′. The higher G′ of SBC-8 surimi paste than the CON was more likely caused by the activation of endogenous TGase by Ca^2+^ ion from ASBB, thereby strengthening the gel network. A higher Gʹ of surimi paste added with nano fishbone powder, compared to the CON, was previously reported [55]. This was coincidental with the increased B-f of the SBC-8 gel.

#### 3.2.3. Scanning Electron Microscopy with Energy Dispersive X-ray Spectroscopy (SEM–EDX)

**Figure 4:**
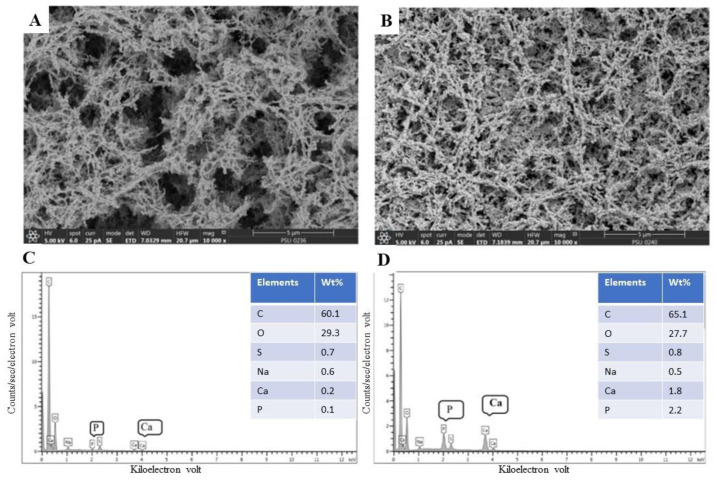
Scanning electron microscope (SEM) with 10,000× magnification and energy dispersive X-ray (EDX) of threadfin bream surimi gel without (CON) ((**A**,**C**), respectively) and with 8% (*w*/*w*) Asian sea bass bio-calcium (SBC-8) ((**B**,**D**), respectively).

SEM-EDX images of both surimi gels representing microstructure and elemental distribution at the surface of surimi gel are shown in Figure 4A–D. In the lack of Ca^2+^ ion, a rougher network with the wider gap or void was noticed in the CON (Figure 4A). This was in accordance with the lower B-f and D-f of the CON (Figure 1). The microstructure of the SBC-8 sample (Figure 4B) showed the denser and finer structure than that of the CON. ASBB with micro size not only increased the crosslinking of protein chains but also filled in the network. Yin and Park [55] documented that the microstructure of surimi gel incorporated with micro fishbone had a dense structure. Surimi gel added with nano fishbone had more compact and denser microstructure when two-step heating was employed than one-step heating [22]. Surimi gel added with CaCl_2_ and whey protein concentrate (WPC) tended to form a finer and denser structure with smaller gaps than that of gel containing WPC [4].

Calcium (Ca), phosphorous (P), sodium (Na), sulfate (S), carbon (C), and oxygen (O) were elements observed in the surface of surimi gel as determined by EDX. No differences in composition of carbon, oxygen, sodium, and sulfate were found between the CON and SBC-8 samples (Figure 4 C,D, respectively). Different peaks were noticed for calcium and phosphorus between both samples. Surimi gel incorporated with 8% (*w*/*w*) ASBB had higher calcium and phosphorus contents. Wijayanti et al. [20] reported that Asian sea bass backbone bio-calcium contained 37.50% (*w*/*w*, dry basis) calcium and 17.40% (*w*/*w*, dry basis) phosphorous.

#### 3.2.4. Proximate Compositions, Calcium, and Phosphorus Contents

Proximate compositions, calcium, and phosphorus contents of the CON and SBC-8 sample are shown in Table 4. Different protein, fat, ash, and carbohydrate contents were observed between both samples (*p* < 0.05). The protein content of the CON was higher than that of the SBC-8 (*p* < 0.05). The CON sample had lower protein content than that of grade SA and grade A white croaker surimi, in which proteins constituted around 62.28–65.54% [54]. Lower protein content was observed in tilapia surimi as sorbitol and sodium tripolyphosphate were added as cryoprotectants [56]. Generally, the protein content of surimi is varied, depending on the species used. Luo et al. [57] reported different protein contents among four different species used for surimi production.

Low fat content was observed in both samples, mainly due to the washing process, in which fat was removed from fish mince. Commonly, the fat content of surimi is less than 1.00% (wet weight basis) [56,58,59] and less than 2.00% (dry weight basis) [60]. A slightly higher fat content was found in the SBC-8 sample. This might be due to the residual fat retained in ASBB to some extent. Wijayanti et al. [20] found that the fat content of Asian sea bass bio-calcium was 0.21%.

Ash content of the SBC-8 sample was greater than that of the CON (*p* < 0.05) as a result of high mineral content in the ASBB added. However, the CON sample had higher ash content than that of surimi from sardine [61], white croaker [60], and tilapia [56]. The higher ash content in the SBC-8 sample (34.18% *w*/*w*, dry basis) than the CON (14.46% *w*/*w*, dry basis) might be governed by some additives incorporated in surimi such as sodium tripolyphosphate or salt, etc. A sharp increase in ash content was found in the SBC-8 sample as a result of the bio-calcium addition. Fish bio-calcium contained high ash representing inorganic matters around 70–90% [20,62,63].

Carbohydrate contents in the CON and SBC-8 sample were 25.73 and 8.17%, respectively. The lower content in the latter was due to the dilution effect by ASBB (8%, *w*/*w*) added into the surimi paste. The high content of carbohydrate in the CON was because of cryoprotectants such as sugar, sorbitol, etc., which were added widely to prevent protein denaturation during the frozen storage.

The SBC-8 sample had higher calcium and phosphorous contents than the CON (*p* < 0.05) due to the abundance of calcium and phosphorous in ASBB. Previous studies reported that calcium and phosphorus of fish bone bio-calcium were around 27–35% and 12–17%, respectively [20,62,63]. Different proportions of calcium and phosphorus between the CON and SBC-8 samples were noticeable. Calcium content of the CON was lower than the phosphorus content. Higher phosphorus content was also owing to the addition of phosphates commonly used in surimi as cryoprotectants or for gel improvement. Polyphosphates play a role in neutral pH retention of the surimi during storage. Thus, myofibrillar protein denaturation during long-term storage might be inhibited or retarded. Sodium pyrophosphate, tetrasodium pyrophosphate, etc. are the phosphates commonly used in surimi [64]. The SBC-8 sample had higher calcium than phosphorus. This directly reflected that ASBB was an excellent source of calcium rather than phosphorus.

## 4. Conclusions

The addition of Asian sea bass bio-calcium (ASBB) increased B-f and D-f, while it reduced EMC of threadfin bream surimi gel. A slightly lower whiteness index was noticed in surimi gel added with ASBB, especially at higher concentrations. Slightly lower intensities of the protein bands (174 kDa) were noticed in samples incorporated with ASBB than the control. Higher likeness scores were attained in the surimi gel added with ASBB, compared to the control. However, a decrease in liking score was observed in surimi gel added with 10% (*w*/*w*) ASBB. Surimi gel added with 8% (*w*/*w*) ASBB had the increased B-f by 80% with the highest likeness score. Higher storage modulus of surimi paste of the sample added with 8% (*w*/*w*) ASBB also confirmed the strengthening role of ASBB in surimi gel formation. Surimi gel added with 8% (*w*/*w*) ASBB exhibited a denser and finer microstructure with higher ash, calcium, and phosphorous content compared to the control. Overall, the addition of bio-calcium from ASBB at 8% (*w*/*w*) improved textural and sensory properties of the surimi gel, and increased the nutritive value, especially calcium and phosphorus.

## Figures and Tables

**Table 1:** Expressible moisture content (EMC), color, and whiteness of threadfin bream surimi gel added with Asian sea bass bio-calcium at different levels.

Samples	EMC (%)	L*	a*	b*	Whiteness
CON	2.42 ± 0.25 ^a^	82.88 ± 0.37 ^e^	−1.08 ± 0.03 ^f^	8.61 ± 0.13 ^f^	80.80 ± 0.35 ^a^
SBC-2	2.01 ± 0.10 ^b^	83.46 ± 0.48 ^d^	−0.74 ± 0.02 ^e^	10.15 ± 0.18 ^e^	80.57 ± 0.36 ^ab^
SBC-4	1.94 ± 0.07 ^b^	84.01 ± 0.27 ^c^	−0.43 ± 0.04 ^d^	11.54 ± 0.15 ^d^	80.27 ± 0.28 ^b^
SBC-6	1.77 ± 0.11 ^bc^	84.25 ± 0.25 ^bc^	−0.14 ± 0.03 ^c^	12.76 ± 0.10 ^c^	79.73 ± 0.23 ^bc^
SBC-8	1.71 ± 0.06 ^c^	84.50 ± 0.24 ^ab^	0.009 ± 0.04 ^b^	13.41 ± 0.17 ^b^	79.50 ± 0.25 ^cd^
SBC-10	1.73 ± 0.04 ^c^	84.76 ± 0.20 ^a^	0.224 ± 0.04 ^a^	14.18 ± 0.11 ^a^	79.18 ± 0.11 ^d^

Values represent mean ± SD (n = 3). Different lowercase superscripts in the same column denote significant differences (*p* < 0.05). CON: Control (without addition of ASBB). SBC-2, SBC-4, SBC-6, SBC-8, and SBC-10: surimi gel added with ASBB (SBC) at 2, 4, 6, 8, and 10% (*w*/*w*), respectively.

**Table 2:** Likeness score of threadfin bream surimi gel added with Asian sea bass bio-calcium at different levels.

Samples	Color	Texture	Odor	Flavor	Taste	Sandy Mouth Feel	Overall
CON	7.44 ± 1.00 ^a^	6.17 ± 0.92 ^c^	6.16 ± 0.95 ^c^	6.58 ± 0.89 ^a^	6.62 ± 0.65 ^a^	6.81 ± 0.88 ^b^	6.31 ± 0.98 ^b^
SBC-2	7.46 ± 0.86 ^a^	6.74 ± 0.92 ^b^	6.58 ± 0.88 ^b^	6.69 ± 0.94 ^a^	6.59 ± 0.84 ^a^	6.78 ± 0.76 ^b^	6.77 ± 0.84 ^a^
SBC-4	7.47 ± 0.85 ^a^	6.72 ± 0.95 ^b^	6.52 ± 0.85 ^bc^	6.61 ± 0.97 ^a^	6.58 ± 0.90 ^a^	7.01 ± 0.92 ^b^	6.81 ± 0.88 ^a^
SBC-6	7.49 ± 0.77 ^a^	6.75 ± 0.81 ^b^	6.95 ± 0.77 ^a^	6.79 ± 0.91 ^a^	6.60 ± 0.90 ^a^	7.17 ± 0.62 ^b^	6.84 ± 0.68 ^a^
SBC-8	7.60 ± 0.65 ^a^	7.39 ± 0.93 ^a^	7.39 ± 0.71 ^a^	7.01 ± 0.77 ^a^	7.08 ± 0.79 ^a^	7.60 ± 0.67 ^a^	7.08 ± 0.81 ^a^
SBC-10	7.50 ± 0.73 ^a^	7.11 ± 0.97 ^a^	7.12 ± 0.90 ^a^	7.01 ± 0.88 ^a^	6.79 ± 0.95 ^a^	6.24 ± 0.91 ^c^	6.52 ± 0.86 ^b^

Values represent mean ± SD (n= 100). Different lowercase superscripts in the same column denote significant differences (*p* < 0.05). CON: Control (without addition of ASBB). SBC-2, SBC-4, SBC-6, SBC-8, and SBC-10: surimi gel added with ASBB (SBC) at 2, 4, 6, 8, and 10% (*w*/*w*), respectively.

**Table 3:** Texture profiles of threadfin bream surimi gel without and with addition of 8% Asian sea bass bio-calcium.

Texture Profiles	CON	SBC-8
Hardness (g)	3508.42 ± 83.09 ^b^	5578.51 ± 53.78 ^a^
Adhesiveness (g s)	−79.84 ± 1.07 ^b^	−136.78 ± 4.12 ^a^
Springiness	8.75 ± 0.08 ^a^	8.82 ± 0.10 ^a^
Cohesiveness	0.56 ± 0.03 ^b^	0.61 ± 0.02 ^a^
Gumminess	2115.86 ± 71.85 ^b^	3394.90 ± 92.16 ^a^
Chewiness	1935.45 ± 60.69 ^b^	3006.77 ± 72.50 ^a^
Resilience	0.25 ± 0.02 ^b^	0.28 ± 0.01 ^a^

Values represent mean ± SD (n = 3). Different lowercase superscripts in the same row denote significant differences (*p* < 0.05). CON: Control (without addition of ASBB). SBC-8: Surimi gel added with 8% ASBB.

**Table 4:** Proximate compositions, calcium and phosphorus contents of threadfin bream surimi gel without and with addition 8% Asian sea bass bio-calcium.

Parameters (%) *	CON	SBC-8
Protein	59.67± 0.04 ^a^	57.45 ± 0.10 ^b^
Fat	0.14 ± 0.01 ^b^	0.19 ± 0.02 ^a^
Ash	14.46 ± 0.56 ^b^	34.18 ± 0.25 ^a^
Carbohydrate	25.73 ± 0.49 ^a^	8.17 ± 0.68 ^a^
Calcium	0.420 ± 0.02 ^b^	6.75 ± 0.05 ^a^
Phosphorus	0.497 ± 0.02 ^b^	5.18 ± 0.10 ^a^

Values represent mean ± SD (n = 3). Different lowercase superscripts in the same row denote significant differences (*p* < 0.05). * Dry weight basis. CON: Control (without addition of ASBB). SBC-8: Surimi gel added with 8% (*w*/*w*) ASBB.

## Data Availability

Not applicable.

## Supplementary Materials

The following are available online at https://www.mdpi.com/article/10.3390/foods10050976/s1, Figure S1: Breaking force (A) and deformation (B) threadfin bream surimi gel added with different levels (0–12%) of ASBB. Different lowercase superscripts denote significant differences (*p* < 0.05). Values represent mean ± SD (n = 3). CON: Control (without addition of ASBB). SBC-2, SBC-4, SBC-6, SBC-8, SBC-10 and SBC-12: surimi gel samples added with ASBB (SBC) at 2, 4, 6, 8, 10 and 12% (*w*/*w*), respectively.

## Abbreviations

*a**	Redness/greenness
AC	Actin
ASBB	Asian sea bass bio-calcium
*b**	yellowness/blueness
B-f	Breaking force
Ca^2+^	Calcium ion
CaCl_2_	Calcium chloride
CaCO_3_	Calcium carbonate
CON	Gel without ASBB
CRD	Completely randomized design
D-f	Deformation
EDTA	Ethylenediaminetetraacetic acid
EMC	Expressible moisture content
Gʹ	Elastic modulus
ICP-OES	Inductively coupled plasma optical emission spectrometer
kDa	Kilodalton
*L**	Lightness
MHC	Myosin heavy chain
NaCl	Sodium chloride
Rf	Relative distances
RT	Room temperature
SBC-2	Surimi gel added 2 %(*w*/*w*) ASBB
SBC-4	Surimi gel added 4 %(*w*/*w*) ASBB
SBC-6	Surimi gel added 6 %(*w*/*w*) ASBB
SBC-8	Surimi gel added 8 %(*w*/*w*) ASBB
SBC-10	Surimi gel added 10 %(*w*/*w*) ASBB
SDS-PAGE	Sodium dodecyl sulfate-polyacrylamide gel electrophoresis
SEM-EDX	Scanning electron microscope-energy dispersive X-ray
SP	Surimi paste
SPSS	Statistical package for social science
TGase	Transglutaminase
TM	Tropomyosin
TN	Troponin
TPA	Texture Profile Analysis
WHC	Water holding capacity
WPC	Whey protein concentrate
