# Supplementary material for: Textural, Sensory, and Chemical Characteristic of Threadfin Bream (Nemipterus sp.) Surimi Gel Fortified with Bio-Calcium from Bone of Asian Sea Bass (Lates calcarifer)
Source: Foods. 2021 Apr 29;10(5):976. doi: 10.3390/foods10050976 (PMC8146884; doi:10.3390/foods10050976)
Supplement: Supplementary file 1 [file foods-10-00976-s001.zip › foods-1191683-supplementary.pdf]

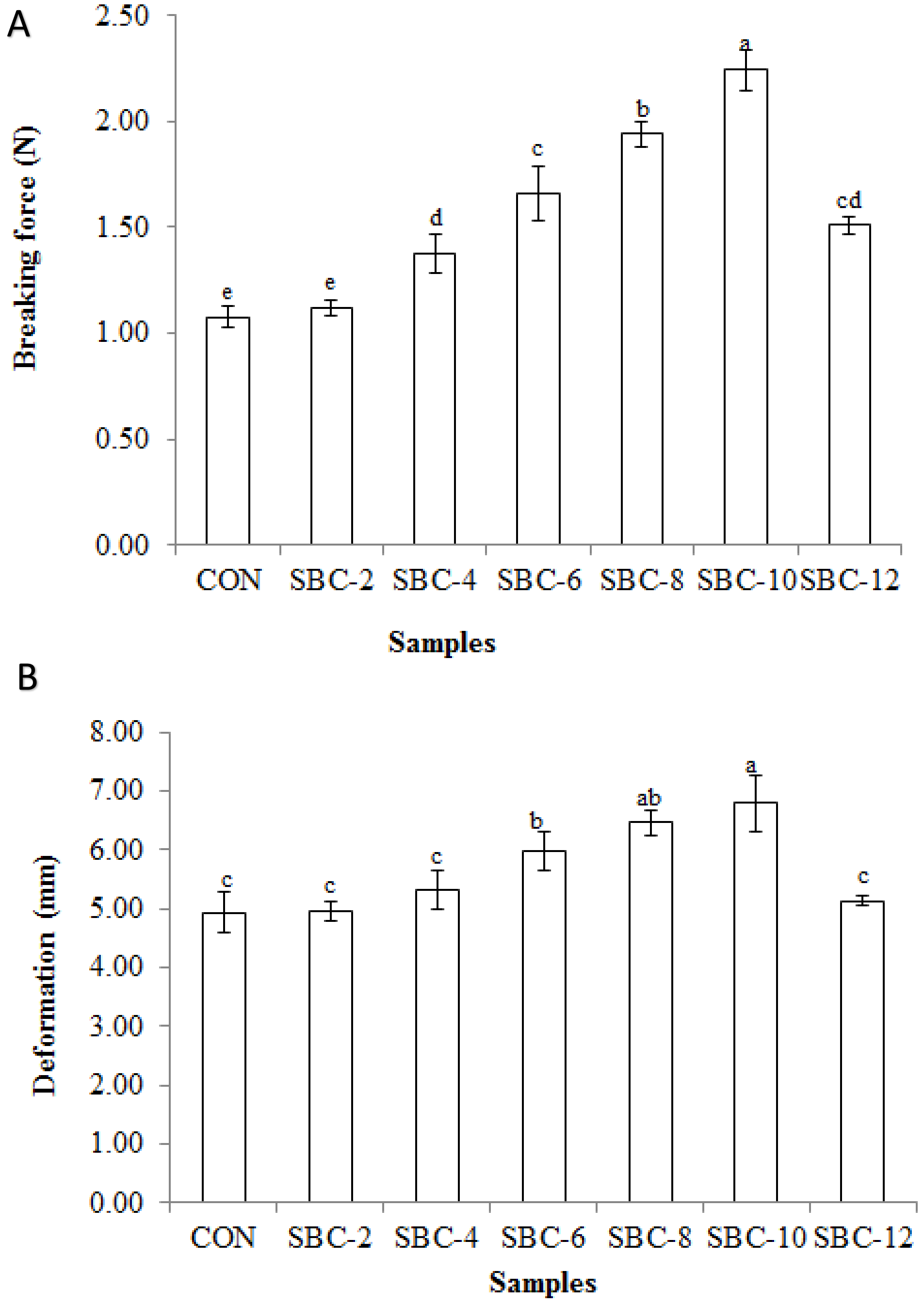

Figure S1. Breaking force (A) and deformation (B) threadfin bream surimi gel added with different levels (0-12%) of ASBB. Different lowercase superscripts denote significant differences ($P < 0.05$). Values represent mean ± SD ($n = 3$). CON: Control (without addition of ASBB). SBC-2, SBC-4, SBC-6, SBC-8, SBC-10 and SBC-12: surimi gel samples added with ASBB (SBC) at 2, 4, 6, 8, 10 and 12% (w/w), respectively.
